# Supplementary material for: Antiviral Immunoglobulins of Chicken Egg Yolk for Potential Prevention of SARS-CoV-2 Infection
Source: Viruses. 2022 Sep 26;14(10):2121. doi: 10.3390/v14102121 (PMC9609661; doi:10.3390/v14102121)
Supplement: Supplementary file 1 [file viruses-14-02121-s001.zip › viruses-1941623-supplementary.pdf]

EVALUATION OF ANTI-SCoV-2 IgY EFFICACY IN SARS-COV-2 INFECTED HAMSTERS

Partial final Report: Study N° 210183

26 September 2022

HEADQUARTERS
18-20 rue Jean Mazen - BP27627
FR-21076 Dijon Cedex
Tel. +33 (0) 380 788 260

SECONDARY ESTABLISHMENT.
25-27 avenue de Québec
FR 91140 Villebon-sur-Yvette Cedex
Tel. +33 (0) 169 296 000

ONCODESIGN Inc.
11-2901 Rue Rachel Est
Montréal, QC H1W 4A4 Canada
Tel. +(1) 514 527 7234

oncodesign.com
contact@oncodesign.com

Study Management

**Testing Company: Oncodesign® Biotechnology**	
Headquarters<br>20 rue Jean Mazen<br>BP 27627<br>21076 Dijon Cedex<br>France<br>☎: +33 (0)3 80 78 82 60<br>✉: contact@oncodesign.com	
**Study Director: Nicolas Legrand, Ph.D., D.V.M.**	
**Signature**	**Date (dd/mm/yy)**
**Associate Study Director: Guillaume Fichet, Ph.D.**	
**Report Writer: Pauline Bornert, PhD, DVM**	

**Sponsor: Norimmun**	
Norimun AS<br>Postboks 469, Sentrum<br>0105, Oslo<br>NORWAY<br>☎: +47 958 50073<br>✉: Lasse@NoriChick.onmicrosoft.com	
**Sponsor representative: Lasse EVENSEN (Project Manager)**	
**Signature**	**Date (dd/mm/yy)**

Experimental Site

Oncodesign Secondary Establishment

Oncodesign Biotechnology
25-27 avenue du Québec
91140 Villebon-sur-Yvette
France
☎: +33 (0)1 69 29 60 00
Site Authorization: Agreement No. B 91 962 106

CEA Fontenay aux Roses (CEA-FAR)

CEA - Commissariat à l'Energie Atomique
18, Route du Panorama
92265 Fontenay-aux-Roses
France
Site Authorization: Agreement No. D92-032-02

Study Summary

Aim
The goal of this study was to evaluate the therapeutic efficacy of anti-RBD-S-SARS-CoV-2/Wuhan IgY antibodies in the COVID-19 golden Syrian hamster model.

Study Timetable	
**Protocol:**	08 July 2021
**Reception of animals:**	13 July 2021
**Randomization:**	19 July 2021
**Treatment Period:**	19 to 22 July 2021
**First animal euthanized:**	23 July 2021
**Last animal euthanized:**	23 July 2021

Protocol	
**Test Substance (batch):**	• Group A: Anti-RBD-IgY (for IN, batch: 12-2020-2)<br>•
**Animals:**	• 18 healthy golden Syrian Hamsters (18 females), 6-8 weeks old at reception, were obtained from Janvier Labs.
**Randomization:**	• Animals were weighed, then allocated into 3 homogenous groups of 6 animals.
**Disease induction:**	• Groups A and B received SARS-CoV-2 on D0 at a dose of 10^5 PFU via the intranasal (IN) route, in a volume of 35µL per nostril (total of 70µL). Group C was not challenged.
**Treatment Schedule:**	• Treatments with test substances were performed following the schedules indicated below:<br>◦ Group A animals (anti-RBD IgY, 4,6 mg) received the test compound by **IN** route (35µL per nostril) **3 times on Day 0 (t-1h, t+1h, t+6h)** and then twice daily **(BID) from Day 1 to Day 3, with an 8h interval** between each delivery. The **first treatment** was performed **1h before infection** with SARS-CoV-2.<br>◦ Group B animals (no test compound) remained unmanipulated before and after infection with SARS-CoV-2.<br>◦ Group C animals received no test compound.
**Monitoring:**	• Body weight was recorded at least once a day after SARS-CoV-2 infection.<br>• A model-specific clinical follow-up was recorded daily after SARS-CoV-2 infection.
**qRT-PCR:**	• Quantification of viral load by RT-qPCR was performed from lung samples taken on D4, using the ORF1ab gene.<br>• Quantification of cytokine gene expression by RT-qPCR was performed from lung samples taken on D4, using the TNFα, IFNγ, IL-2, IL-4, IL-6, IL-10, IL-12p40 and IL-21 genes.

**Virus $TCID_{50}$:**	• Viral infectious particle titration using samples collected on D4 was performed using $TCID_{50}$ on Vero E6/TMPRSS2 cells.
**Lung histology:**	• Lung samples collected on D4 were scored for histomorphometric changes.
**Results**	
**Health Parameters:**	From D1 to D4, the non-challenged animals from Group B maintained a stable body weight. Conversely, challenged non-treated animals from Group C continuously lost weight after the viral challenge, reaching a mean of 91.5% of D0 body weight on D4. Animals from Group B lost a little less weight throughout the course of the in vivo phase, reaching respectively 95.8% and 95.33% of D0 body weight on D4.<br><br>Statistical tests showed a significant effect of group on weight change throughout the 4 days of the study. On D4, there was a significant difference in body weight loss between Groups A and C, and B and C (Table S5).
**Efficacy:**	Mean levels of viral RNA were approximately 3-fold lower in treated group A than in Challenged non-treated group B on D4. The differences were statistically significant (Table S7).<br><br>Treatments were associated with a small decrease in $TCID_{50}$ (less than a 2-fold). However, these differences were not statistically significant (Table S9).<br><br>IL-6 expression was strongly induced by the viral challenge as well: expression levels were higher in Group B than C (statistically significant, Table S13). Expression levels were also higher in treated Group A than in non-challenged Group C.<br><br>IL-10 expression was strongly induced by the viral challenge with higher expression levels in Group B than C (statistically significant, Table S13). Expression levels were also higher in treated Group A than in non-challenged Group C. Expression Il-2, Il-4, IL-12p40 and IL-21 was not different across groups, including between challenged and non-challenged groups (no significant difference, Table S13).
**Conclusions**	

- No signs of toxicity of the treatments on body weight were observed, compared to the non-treated challenged group (Group B).
- On D4 RBD-IgY (Group A was associated with a reduction in viral load measured by qRT-PCR in the lungs of the challenged animals, compared to the non-treated challenged animals. In coherence with that, a small non-significant reduction of viral infectious particles measured by $TCID_{50}$ was observed in these groups.

Table of Contents

Study Management 2

Experimental Site 3

Study Summary 4

Table of Contents 6

List of Figures .... **Error! Bookmark not defined.**

List of Tables 8

List of Abbreviations 9

1 Study Aim 10

2 Materials and Methods 10

2.1 Use of animals 10

2.1.1 Ethical statement 10

2.1.2 Housing conditions 10

2.2 SARS-CoV-2 virus 10

2.3 Test and reference substances 11

2.3.1 Test substance 11

2.3.2 Vehicle 11

3 Experimental Design and Treatments 11

3.1 Overview 11

3.2 Animals 12

3.3 Randomization 12

3.4 Treatment schedule 12

3.5 SARS-CoV-2 challenge (D0) 12

3.6 Euthanasia of animals on D4 (4DPI) 12

3.7 Sample list summary 13

4 Ex vivo analysis 13

4.1 Virus load in lungs by genomic qRT-PCR (D4) 13

4.2 Virus $TCID_{50}$ in lungs (Day 3) 13

4.3 Cytokine profiling in lungs by qRT-PCR (Day 4) 14

4.4 Lung histology (Day 4) 15

5 Animal monitoring 16

5.1 Clinical monitoring 16

5.2 Humane endpoints 16

5.2.1 Anesthesia and analgesia 17

5.2.2 Euthanasia and necropsy 17

6 Data Presentation and Management 17

6.1 Quality Assurance 17

6.2 Health parameters 17

6.3 Efficacy parameters ... 17
6.4 Statistical tests ... 17
7 Deviations to the study protocol ... **Error! Bookmark not defined.**
8 Results ... 17
8.1 Randomization ... 17
8.2 Health parameters ... 17
8.3 Efficacy Parameters ... 18
8.3.1 Viral RNA expression in the lungs ... 18
8.3.2 Viral load in the lungs determined by $TCID_{50}$ ... 18
8.3.3 Lesion score in the lungs ... 18
8.3.4 Cytokine expression in the lungs ... 18
9 Conclusions ... 18
10 Archiving ... 19
11 Confidential Disclosure Agreement ... 19
12 Bibliography ... 19
13 Figures, Tables and Statistical Analyses ... 20
13.1 Randomization ... 20
13.2 Health Parameters ... **Error! Bookmark not defined.**
13.3 Efficacy Parameters ... **Error! Bookmark not defined.**
Appendix A: Raw data ... 34
14 Raw Data List ... 35
14.1 List of full group names ... 35
Appendix B: Protocol ... **Error! Bookmark not defined.**
Appendix C: Animal Health Report ... **Error! Bookmark not defined.**
Appendix D: Test substance information ... **Error! Bookmark not defined.**
Appendix E: Quality assurance statement ... **Error! Bookmark not defined.**

List of Tables

Table S1: Summary of mean body weight (g) on the day of randomization (D-3) of golden Syrian hamsters inoculated with SARS-CoV-2. 20

Table S2: ANOVA test of the randomization criteria on D-3. 20

Table S3: Summary of body weights expressed in grams (g) of golden Syrian hamsters. 21

Table S4: Summary of body weights change expressed in % of D0 of golden Syrian hamsters. 22

Table S5: Two-way ANOVA of the mean body weight change between D0 and D4 23

Table S6: Viral RNA expression level (mean and SD) in lung from golden Syrian hamsters. 24

Table S7: ANOVA of viral RNA expression level in lung from golden Syrian hamsters. 25

Table S8: Viral load determined by $TCID_{50}$ (mL/g of lung, mean + SD) in lung from golden Syrian hamsters. 26

Table S9: ANOVA of viral load by TCID50 in lung from golden Syrian hamsters. 27

Table S10: Summary of the lesion score in the lungs of golden Syrian hamsters. 28

Table S11: ANOVA of the lesion scores in lung from golden Syrian hamsters. 29

Table S12: Quantitation of the cytokine expression levels in the lungs of golden Syrian hamsters expressed as $2^{-\Delta ct}$. 30

Table S13: ANOVA on the cytokine expression levels in the lungs of golden Syrian hamsters. 32

List of Abbreviations

Adm	Administration
ANOVA	Analysis of variance
BW	Body weight
DR	Day of randomization
DX	Day of measurement
FD	Found dead
IN	Intranasal
IP	Intraperitoneal(ly)
IV	Intravenous
NA	Not applicable
ND	Not determined
N°	Number of
NS	Not significant
p	Probability
Ref	Reference
SC	Subcutaneous(ly)
SD	Standard deviation
Sign	Significant
V	Volume
vs	Versus

1 Study Aim

The goal of the present study was to evaluate the therapeutic efficacy of IgYs developed by Norimmun against SARS-CoV-2 in the COVID-19 golden Syrian hamster model.

2 Materials and Methods

2.1 Use of animals

2.1.1 Ethical statement

Animal housing and experimental procedures were conducted according to the French and European Regulations and the National Research Council Guide for the Care and Use of Laboratory Animals [1], [2]. The animal facility is authorized by the French authorities (CFH: Agreement N° B 91 962 106). All procedures on non-infected animals (including surgery, anesthesia and euthanasia as applicable) used in the current study are submitted to the Institutional Animal Care and Use Committee of Oncodesign (Oncomet) approved by French authorities [CNREEA agreement N° 91 (Oncodesign)].

The animal BSL3 facility is authorized by the French authorities (Agreement N° D92-032-02). All procedures on SARS-CoV-2 infected animals (including surgery, anesthesia and euthanasia as applicable) used in the current study (Ethical protocol: CEA-FAR APAFIS #27637-2020101209323274 v1) are submitted to the Institutional Animal Care and Use Committee of CEA approved by French authorities (CETEA DSV – n° 44).

2.1.2 Housing conditions

Animals were maintained in specific-pathogen free health status according to the Federation for Laboratory Animal Science Associations guidelines. Animals were individually identified. Animals were maintained in housing rooms under controlled environmental conditions:

- temperature: 22 ± 2°C,
- humidity 55 ± 10%,
- photoperiod (12h light and 12h dark),
- H14 filtered air
- minimum of 12 air exchanges per hour with no recirculation.

Each cage was labeled with a specific code. Animal enclosures provided sterile and adequate space with bedding material, food and water, environmental and social enrichment (group housing) as described below:

- IsoRat900N biocontainment system (Tecniplast, France),
- Poplar bedding (Select fine, Safe, France),
- A04 SP-10 diet (Safe, France),
- Tap water
- Environmental enrichment
    - Tunnel
    - Wood sticks

2.2 SARS-CoV-2 virus

- SARS-CoV-2 strain "Slovakia/SK-BMC5/2020", originally provided by the European Virus Archive global (EVAg) (GISAID EPI_ISL_417879, https://www.european-virus-archive.com/virus/sars-cov-2-strain-slovakiask-bmc52020), produced and tittered by Oncodesign on VeroE6-TMPRSS2 cells, was used for hamster infection. The strain belongs to the GH clade.
- Virus production was performed in T175 flasks seeded with $50x10^6$ VeroE6-TMPRSS2 cells and in a 40mL final volume. Cell counts and viability were assessed by 0.25% trypan blue exclusion assay by ViCell apparatus. After 48hrs of infection time frame (with 0.001-0.005 MOI of SARS-CoV-2 virus), cytopathogenic effects were confirmed under microscope observation. Culture supernatant was harvested, centrifuged (5min at 5000g) and aliquoted (1mL aliquots).
- Virus stock $TCID_{50}$ titers were determined on VeroE6-TMPRSS2 cells. About two hours before testing, cells were plated in 96-well plate at the density of $2x10^4$ cells per well in a volume of 200 µL of complete growth medium (DMEM 10% FCS). Cells were infected with serial dilutions of virus stock (8-plicates; 1st

dilution 1:100; 5-fold serial dilutions) for 1h at 37°C. Fresh medium was added for 48 hours and a MTS-PMS assay was then performed, according to provider protocol (Promega, reference #G5430). Plates were read using an ELISA Plate reader and the data was recorded. Infectivity is expressed as $TCID_{50}$/mL/48h based on the Spearman-Karber formula.

2.3 Test and reference substances

2.3.1 Test substance

Test substances listed in the table below were provided by the sponsor.

Test substance	Batch	Concentration - Quantity (unit)	Storage
Group A: Anti-RBD-IgY (for IN)	12-2020-2	65.7 mg/mL - 7 mL	2-8°C

All remaining test substances were destroyed at the end of the study.

- ✓ The test substance anti-CoV-2 IgY was provided as a ready-to-use liquid. For group A, B and D, IgY-aliquots were shaken before use and volume extracted from the tubes was used directly for IN administration. No dilution was required. For group C, IgY-solution was diluted 60x in drinking water (150mL water bottles replaced daily and prepared with 2.5mL of test compound + 147.5mL H_2O).
- ✓ **Storage:** The test item was stored at 2-8°C.
- ✓ **Preparation and use:** Inoculations was performed after dilution in the study vehicle.

- ✓ The test substance pegIFNα2a was provided as a ready-to-use liquid (reference: 008767, PEGASYS) in syringes (135µg/0.5mL each).
- ✓ **Storage:** The test item was be stored at 2-8°C.
- ✓ **Preparation and use:** Inoculations were performed after dilution in the study vehicle.

2.3.2 Vehicle

- ✓ The formulation vehicle was provided by Oncodesign.
- ✓ **Standard Vehicle:** Sodium chloride 0.9% (Osalia 100mL).

3 Experimental Design and Treatments

3.1 Overview

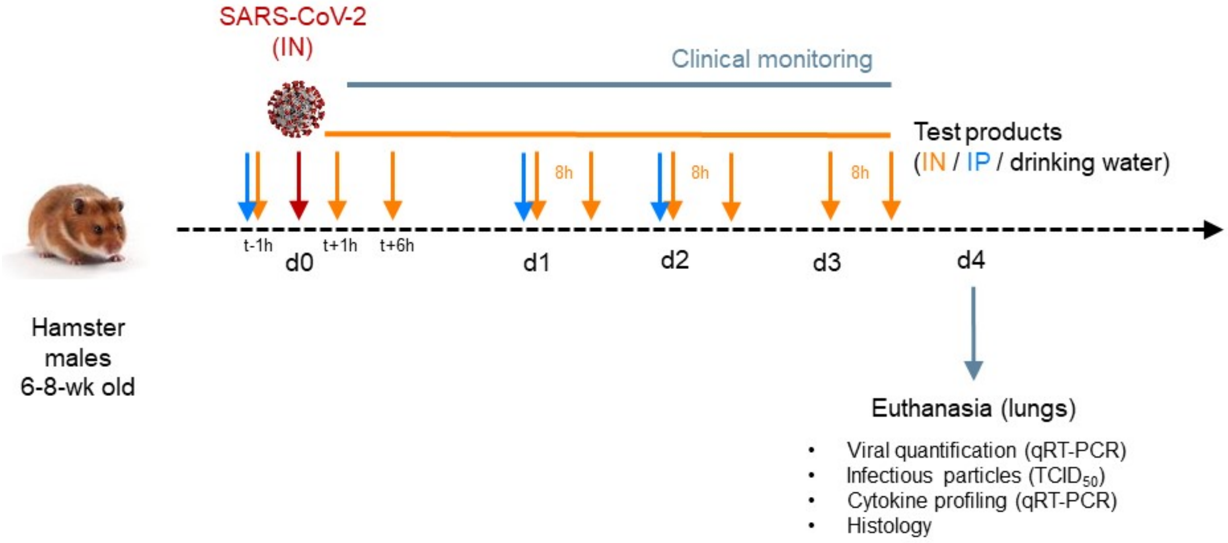

3.2 Animals

Healthy golden Syrian Hamsters (18 females), 6-8 weeks old at reception, were obtained from Janvier Labs.

3.3 Randomization

Animals were weighed, then allocated into 3 homogenous groups of 6 animals. Animals were labeled on the skin back (1cm^2 shaved area) using animal-dedicated pencils.

3.4 Treatment schedule

✓ The administration route of the test substances had been chosen by the sponsor.
  - The test substance anti-CoV-2 IgY was either delivered using the intranasal (IN) route, or in the drinking water, without anesthesia. Anesthesia cannot be applied more than once a day.
  - The test substance pegIFNα2a was delivered using the intraperitoneal (IP) route).
  - The first treatment was performed 1h before infection with SARS-CoV-2.

✓ Treatments with test substances were performed following the schedules indicated below:
  - Group A animals (anti-RBD IgY, 4,6 mg) received the test compound by **IN** route (35µL per nostril) **3 times on Day 0 (t-1h, t+1h, t+6h)** and then twice daily **(BID) from Day 1 to Day 3**, **with an 8h interval** between each delivery. The **first treatment** was performed **1h before infection** with SARS-CoV-2.
  - Group B animals (no test compound) remained unmanipulated before and after infection with SARS-CoV-2.
  - Group C animals (no test compound) remained fully unmanipulated (no SARS-CoV-2 infection).

The treatment schedule is summarized in the table below:

Group	No. animals	Test	Treatment schedule	Dosage/ injection	Total quantity*	Virus (IN, d0)	End point
A<br>RBD-IgY	6	anti-CoV-2 IgY	3x on D0 (IN)<br>(t-1h/+1h/+6h)<br>+ BID (8h delay; IN) (D1/2/3)	4.6 mg<br>(35µL/nostril)	323 mg	10^5 pfu	d4
B	6	No	N.A.	N.A.	N.A.	10^5 pfu	d4
C	6	No	N.A.	N.A.	N.A.	No	d4

IN: intra-nasal; IP: intraperitoneal. * including a minimal 30% test product excess (average hamster weight of 120g).

3.5 SARS-CoV-2 challenge (D0)

- All the animals from groups A and B (total n=30) received SARS-CoV-2 on D0.
- Group C animals (n=6) remained unchallenged.
- The administration route of SARS-CoV-2 had been chosen by Oncodesign. The treatment was administered by intranasal route (IN) under a total volume of 70µL (35µL per nostril) on Isoflurane-anesthetized animals. An intranasal dose of 10^5 PFU per animal was administered.

3.6 Euthanasia of animals on D4 (4DPI)

- The infected animals of all study groups A/B and unchallenged animals of group C, (n=6 animals per group, total n=18) were terminated on Day 4 post-infection.
- Animals were deeply anesthetized using a cocktail of Zoletil (30mg/kg – 0.6mL/kg) and xylazine (10mg/kg – 0.5mL/kg) injected by intraperitoneal route. Gentle cervical dislocation followed by thoracotomy were performed before lung collection.

- Superior right lobe was put in RNA Later overnight at 4°C, then stored at -80°C until RNA extraction for quantification of viral load by qRT-PCR.
- Middle, post-caval and inferior right lobes were snap frozen in liquid nitrogen (one lobe per tube), then stored at -80°C until quantification of viral infectious particles ($TCID_{50}$).
- Left lungs were put in formalin for histology for at least 24 hours, followed by paraffin embedding. The weight of the lobe was recorded at time of sample transfer into the formalin tube (difference between tube weight without vs. with sample).

3.7 Sample list summary

- The following table provides an overview of the harvested samples in the course of the study.

Study Day	Date	Group / Number of animals	Action / Procedure / Samples<br>(animal related activity other than routine)	Number of samples
D0	19-Jul-2021	Groups A-B / 12	SARS-CoV-2 challenge	-
D4	23-Jul-2021	Groups A-B-C / 18	Endpoint 4dpi<br>(terminal lung harvest)	18 lung series

4 Ex vivo analysis

4.1 Virus load in lungs by genomic qRT-PCR (D4)

- Quantification of viral load by RT-qPCR was done from lung using viral ORF1ab gene.
- Extraction of viral RNA was performed using the Macherey Nagel NucleoSpin 96 RNA, 96-well kit for RNA purification (ref. #740709.4). RNA was frozen at -80°C until RT-qPCR.
- RT was performed with the High Capacity cDNA Reverse Transcription Kit from Applied Biosystem (ref. # 4368813).
- cDNA quantification by real time quantitative PCR was performed with primers conditions targeting ORF1ab gene. Amplifications was performed using a QuantStudio 7 Flex from Applied Biosystem and adjoining software.
- SYBR Green technology was used for PCR product detection & quantification (PowerSYBR green PCR Master Mix – AppliedBiosystems, ref. #4367659).

**Primers and Probes**	
Name	Sequences (5'-3')
ORF1ab gene nCoV	
ORF1ab_Fw	CCGCAAGGTTCTTCTTCGTAAG
ORF1ab_Rv	TGCTATGTTTAGTGTTCCAGTTTTC

4.2 Virus $TCID_{50}$ in lungs (D4)

The tissue culture infective dose that causes 50% cytotoxicity ($TCID_{50}$) assay is a quantitative method for assessing the infectivity of a virus stock. One $TCID_{50}$ is defined as the amount of pathogen that causes death of 50% of cells (Reed and Muench, 1938), so $TCID_{50}$ depends on the ability of the virus to kill the cells in culture. Infectivity is expressed as $TCID_{50}$/mL/48h based on the Spearman-Karber formula.

- VeroE6-TMPRSS2 cells were counted and their viability was assessed by 0.25% trypan blue exclusion assay by ViCell apparatus.
- One day before testing, cells were plated in a 96-well plate at the density of $2x10^4$ cells per well in a volume of 200 µL of complete growth medium (DMEM 10% FCS).

- Cells were infected with serial dilutions of the lung homogenate (triplicate) for 1h at 37°C. Fresh medium was added for 48 hours
- 48 hours after cell infection, an MTS-PMS assay was performed. Assay was performed according to provider protocol (Promega ref#G5430). After discarding all supernatant, 100µL of fresh medium and 20µL of MTS-PMS reagent are added to the culture wells. After a maximum of 4 hours, plates were read using an ELISA Plate reader and data was recorded (OD value in negative cell control > 1.500).

4.3 Cytokine profiling in lungs by qRT-PCR (D4)

✓ Cytokine gene expression in lungs was determined for 8 target genes:
TNFα
IFNγ
IL-2
IL-4
IL-6
IL-10
IL-12p40
IL-21

✓ Primer sequences for each target gene are provided in the following table:

**Primers and Probes**	
**Name**	**Sequences (5'-3')**
**TNFα**	Fw : TGAGCCATCGTGCCAATG<br>Rv : AGCCCGTCTGCTGGTATCAC<br>Probe : 5'-(6FAM)-CGG CAT GTC TCT CAA AGA CAA CCA G-(TAMRA)-3
**IFNγ**	Fw : TGTTGCTCTGCCTCACTCAGG<br>Rv : AAGACGAGGTCCCCTCCATTC<br>Probe : 5'-(6FAM) TGG CTG CTA CTG CCA GGG CAC ACT C-(TAMRA)-3'
**IL-2**	TBD
**IL-4**	Fw : ACAGAAAAAGGGACACCATGCA<br>Rv : GAAGCCCTGCAGATGAGGTCT<br>Probe : 5'-(6FAM) AGA CGC CCT TTC AGC AAG GAA GAA CTC C-(TAMRA)-3'
**IL-6**	Fw : AGACAAAGCCAGAGTCATT<br>Rv : TCGGTATGCTAAGGCACAG<br>Probe : TBD
**IL-10**	Fw : GGTTGCCAAACCTTATCAGAAATG<br>Rv : TTCACCTGTTCCACAGCCTTG<br>Probe : 5'-(6FAM) TGC AGC GCT GTC ATC GAT TTC TCC C-(TAMRA)-3'
**IL-12p40**	Fw : AATGCGAGGCAG CAAATTACTC<br>Rv : CTGCTCTTGACGTTGAACTTCAAG<br>Probe : 5'-(6FAM)-CCT GCT GGT GGC TGA CTG CAA TCA-(TAMRA)-3'
**IL-21**	Fw : GGACAGTGGCCCATA AAACAAG<br>Rv : TTCAACACTGTCTATAAGATGACGAAGTC<br>Probe : 5'-(6FAM)-CAA GGG CCA GAT CGC CTC CTG ATT-(TAMRA)-3'

4.4 Lung histology (D4)

Left lung specimens harvested at euthanasia were embedded in paraffin (1 slide by animal). Sections of 5 µm thickness were cut and mounted on SuperFrost plus glass slides, and stained with H&P (Hematoxylin-Phloxin) to visualize histomorphometric changes. Slides were scanned using the NanoZoomer Digital Pathology System C9600-02, and analyzed using Definiens software.

For each section, several criteria were evaluated:

1. Recruitment of inflammatory cells for bronchial and alveolar walls;
2. Presence of pulmonary edema;
3. Presence of alveolar hemorrhage;

A scoring grid was used as follows:

Score	Inflammation	Edema	Hemorrhage
0	no pathological changes	Absence	Absence
1	affected area <= 10%	Presence	Presence
2	affected area >10% to <50%		
3	affected area >= 50%		

Examples of images and corresponding scoring are provided below for each criterion.

1. Inflammation

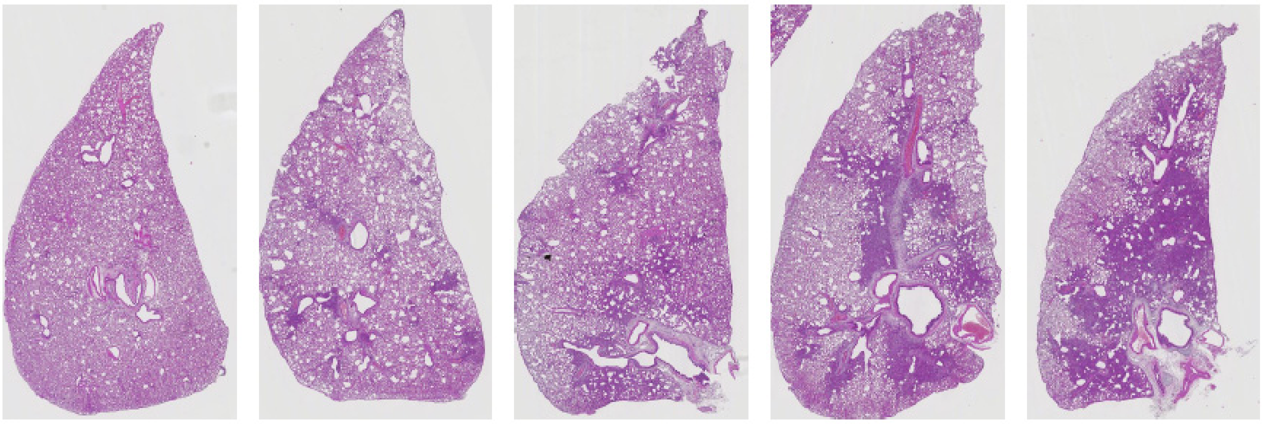

No inflammation <10% 10-50% >50%

2. Pulmonary edema

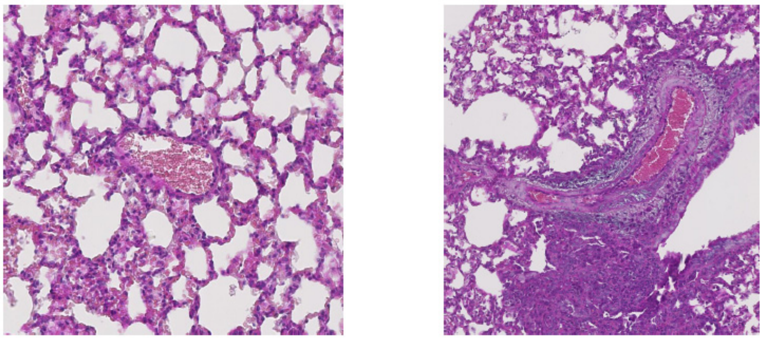

Absence Presence

3. Alveolar hemorrhage

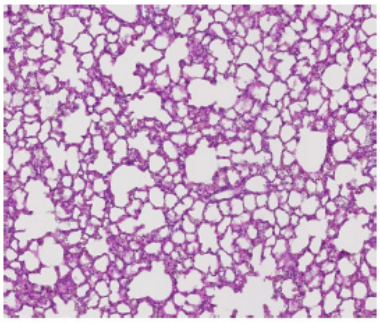

Absence

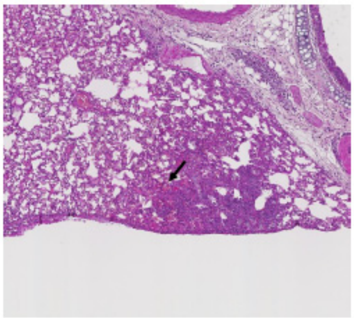

Presence

5 Animal monitoring

5.1 Clinical monitoring

Animal viability, behavior and body weight were monitored daily after SARS-CoV-2 infection.

In the context of infection with the Slovakia/SK-BMC5/2020 SARS-CoV-2 strain, body weight is the only relevant clinical sign.

Historical accumulation of data obtained from daily clinical follow-up after SARS-CoV-2 infection did not show any significant clinical score, using the listed parameters below:

- Body weight loss
- Piloerection (absence – slight – marked)
- Behavior (normal – less mobile – amorphous or isolated)
- Posture (normal – abnormal – hunched)
- Cough (presence or absence)
- Sneeze (presence or absence)

5.2 Humane endpoints

Humane endpoints requiring specific action had been established as follows:

- Subcutaneous rehydration with Ringer Lactate would be performed in case of a body weight loss ≥ 15% (compared to a reference day e.g. the first day of treatment). If transient toxicity were to be suspected, treatment would be stopped.
- If dehydration were to be observed, animals would be rehydrated with fluid or provided with hydrogel.

Humane endpoints requiring euthanasia [3], [4] had been established as follows:

- Twenty percent body weight loss (compared to the first day of treatment or maximum weight) lasting for a maximum of two consecutive days,
- Signs of pain, suffering or distress: pain posture, pain face mask, abnormal behavior or vocalization,
- Poor body condition, emaciation, cachexia, dehydration,
- Bladder outflow obstruction or diarrhea over a 48h period,
- Prolonged absence of voluntary responses to external stimuli,
- Rapid labored breathing,
- Anemia, significant bleeding,
- Bloodstained or mucopurulent discharge from any orifice,
- Neurologic signs: circling, convulsion, hind limb paralysis,
- Sustained decrease in body temperature,
- Abdominal distension.

5.2.1 Anesthesia and analgesia

Isoflurane gas anesthesia was used for IN infections and treatment administrations.

If necessary, non-pharmacological care, such as rehydration or gel diet, would have been provided. Additionally, pharmacological care that did not interfere with the study would have been provided at the recommendation of the attending veterinarian.

5.2.2 Euthanasia and necropsy

Euthanasia of animals was performed under deep anesthesia using a cocktail of Zoletil (30mg/kg – 0.6mL/kg) and xylazine (10mg/kg – 0.5mL/kg) injected by intraperitoneal route followed by exsanguination (if required) and cervical dislocation. If physical methods for euthanasia (cervical dislocation) were necessary, they were performed by highly skilled and trained technicians.

Necropsy (macroscopic examination) was performed on all animals euthanized in the study, and, if possible, on all euthanized moribund animals; and those found dead.

6 Data Presentation and Management

The raw data was provided to the study sponsor as MS Excel® files after the end of the in vivo phase, as soon as the data became available.

6.1 Quality Assurance

Internal quality control checking was performed on raw data and worksheets during and after completion of this study and documented by a dated signature with statement to the effect that the data are accurate.

6.2 Health parameters

- Individual and mean body weight of animals were provided,
- Individual and mean clinical scores of animals were provided.

6.3 Efficacy parameters

- Individual and mean lung viral load determined by RT-qPCR were provided
- Individual and mean lung viral infectious particles determined by $TCID_{50}$ were provided
- Individual and mean lung cytokine expression level determined by RT-qPCR were provided
- Individual and mean lung histopathology analysis were provided.

6.4 Statistical tests

GraphPad Prism® (GraphPad Prism Software, USA) was used for preparation of the tables, figures and statistical analyses. Statistical analyses comparisons were performed using an ANOVA for analyses between all groups. A p value ≤ 0.05 was considered significant.

7 Results

7.1 Randomization

On the day of randomization (D-3), mean body weight was 90.89g (Table S1). Statistical analysis showed no significant differences between groups (Table S2).

7.2 Health parameters

Animal body weight and clinical scores were monitored throughout the study. Clinical scores remained equal to zero, with the animals showing no clinical signs. Thus, the only relevant health parameter was body weight (Table S3). Body weights, expressed as a percentage of body weight compared to the weight on D0, are shown in **Error! Reference source not found.** and Table S4.

From D0 to D4, the non-challenged animals from Group C maintained a stable body weight. Conversely, challenged and non-treated animals from Group B continuously lost weight after the viral challenge, reaching a mean of 91.5% of D0 body weight on D4. Animals from Group A lost a little less weight throughout the course of the in vivo phase, reaching respectively 95.8% and 95.33% of D0 body weight on D4.

Statistical tests showed a significant effect of the group on weight change throughout the 4 days of the study. On D4, there was a significant difference in body weight loss between Groups A and C,, and B and C (Table S5).

7.3 Efficacy Parameters

7.3.1 Viral RNA expression in the lungs

Animals were inoculated with the viral strain on D0 and treated on from D0 to D3. Quantification of the viral gene ORF1ab and of a housekeeping gene (β actin) was performed using RT-qPCR on lung samples collected from the animals on D4 (**Error! Reference source not found.** and Table S6). Results are expressed as a relative expression level between the two genes ($2^{-\Delta CT}$).

Mean levels of viral RNA were approximately 3-fold lower in treated group A than in challenged non-treated group B on D4. The differences were statistically significant (Table S7).

7.3.2 Viral load in the lungs determined by $TCID_{50}$

Lung viral load (in number of infectious viral particles per g of lung) was determined from virus titers based on samples taken from the animals on D4. The results are summarized in **Error! Reference source not found.** and Table S8.

7.3.3 Lesion score in the lungs

Lesion scoring was performed based on histomorphometric analysis of lung samples (inflammation, edema and hemorrhage) collected from the animals on D4 (**Error! Reference source not found.** and Table S10).

7.3.4 Cytokine expression in the lungs

Quantification of the expression of cytokine genes (TNFα, IFNγ, IL-2, IL-4, IL-6, IL-10, IL-12p40 and IL-21) and of a housekeeping gene (β actin) was performed using RT-qPCR on lung samples collected from the animals on D4 (**Error! Reference source not found.** and Table S12). Results are expressed as a relative expression level between the two genes ($2^{-\Delta CT}$).

IFNγ expression was strongly induced by the viral challenge, as expression levels were much higher in Group B than C (statistically significant, Table S13). Expression levels were also higher in treated Group A than in non-challenged Group C.

IL-6 expression was strongly induced by the viral challenge as well: expression levels were higher in Group B than C (statistically significant, Table S13). Expression levels were also higher in treated Group A than in non-challenged Group C. Additionally, no treated Group exhibited significantly different IL-6 expression compared to Group D (challenged, non-treated).

IL-10 expression was strongly induced by the viral challenge with higher expression levels in Group B than C (statistically significant, Table S13). Expression levels were also higher in treated Group A than in non-challenged Group C.

Expression Il-2, Il-4, IL-12p40 and IL-21 was not different across groups, including between challenged and non-challenged groups (no significant difference, Table S13).

8 Conclusions

- No signs of toxicity of the treatments on body weight were observed, regardless of the route of administration, compared to the non-treated challenged group (Group B).
- On D4, treatments with RBD-IgY (Group B were associated with a reduction in viral load measured by qRT-PCR in the lungs of the challenged animals, compared to the non-treated challenged animals. In

coherence with that, a small non-significant reduction of viral infectious particles measured by $TCID_{50}$ was observed in these groups.

- On D4, IFNγ, IL-6, and IL-10 expression had been induced in the challenged non-treated groups compared to the non-challenged group (Group C). No treated group exhibited differential lung expression of IFNγ or IL-6 compared to the non-treated challenged group (Group B). Expression of Il-2, Il-4, IL-12p40 and IL-21 was not significantly induced by the viral challenge.

9 Archiving

Oncodesign will conserve documents, reagents, and samples for a duration of 3 years after the end of the study. The end of the study is defined as the date of the signature of the report by the study director.

At the end of this period, the archived content will be destroyed. Alternatively, the archives can be returned to the sponsor if specifically requested, at their expense.

10 Confidential Disclosure Agreement

The sponsor shall have the exclusive ownership of the results of the studies and research subject to the present agreement. Oncodesign agrees to maintain in confidence all confidential information. It is forbidden for Oncodesign to publish or to communicate any results without the written agreement of the sponsor. The results of the study can be freely used by the sponsor, for example for public presentations, documentation, marketing authorizations, publications, information for physicians and pharmacists, etc.

11 Bibliography

[1] Parlement européen et du Conseil du 22 septembre 2010. (2013, février 01)., *Décret n° 2013-118 du 1er février 2013 relatif à la protection des animaux utilisés à des fins scientifiques*.

[2] National Research Council (U.S.), Institute for Laboratory Animal Research (U.S.), and National Academies Press (U.S.), Eds., *Guide for the care and use of laboratory animals*, 8th ed. Washington, D.C: National Academies Press, 2011.

[3] P. Workman *et al.*, “Guidelines for the welfare and use of animals in cancer research,” *Br. J. Cancer*, vol. 102, no. 11, pp. 1555–1577, May 2010, doi: 10.1038/sj.bjc.6605642.i

[4] Organisation For Economic Co-Operation And Development, “OECD Environmental Health and Safety Publications Series on Testing and Assessment No. 19. Guidance document on the recognition, assessment, and use of clinical signs as humane endpoints for experimental animals used in safety evaluation.” Nov. 2000.

12 Figures, Tables and Statistical Analyses

12.1 Randomization

Table S1: Summary of mean body weight (g) on the day of randomization (D-3) of golden Syrian hamsters inoculated with SARS-CoV-2. Animals were randomized on D-3, inoculated with the viral strain on D0 and treated from D0 to D3. D4 was the last day of the study**.**

Group	n	Mean	SD	Minimum	Maximum
**Group A - RBD-IgY IN**	6	90.50	4.64	86	98
**Group B - non-treated**	6	90.67	6.98	80	97
**Group C - non-challenged**	6	91.83	5.12	86	99

Table S2: ANOVA test of the randomization criteria on D-3.

ANOVA table	SS	DF	MS	F (DFn, DFd)	P value
**Treatment (between columns)**	23.56	5	4.711	F (5, 30) = 0.1375	P=0.9823
**Residual (within columns)**	1028	30	34.27		
**Total**	1052	35			

Table S3: Summary of body weights expressed in grams (g) of golden Syrian hamsters. Animals were randomized on D-3, inoculated with the viral strain on D0 and treated from D0 to D3. D4 was the last day of the study.

**Group**		**Study Days**				
		**0**	**1**	**2**	**3**	**4**
**Group A - RBD-IgY IN**	**mean**	95.00	94.17	91.50	88.83	88.83
	**SD**	5.48	5.23	5.61	5.64	5.49
	**n**	6	6	6	6	6
**Group B - non-treated**	**mean**	96.67	94.50	91.67	90.67	88.67
	**SD**	6.98	7.40	7.42	7.31	7.76
	**n**	6	6	6	6	6
**Group C - non-challenged**	**mean**	95.33	94.67	94.50	95.67	94.00
	**SD**	5.20	6.15	7.42	6.77	8.29
	**n**	6	6	6	6	6

Table S4: Summary of body weights change expressed in % of D0 of golden Syrian hamsters. Animals were randomized on D-3, inoculated with the viral strain on D0 and treated from D0 to D3. D4 was the last day of the study.

**Group**		**Study Days**				
		**0**	**1**	**2**	**3**	**4**
**Group A - RBD-IgY IN**	**mean**	100.00	99.17	96.33	93.33	93.33
	**SD**	0.00	1.47	1.86	2.80	3.01
	**n**	6	6	6	6	6
**Group B - non-treated**	**mean**	100.00	97.83	94.83	93.67	91.50
	**SD**	0.00	1.83	3.54	3.98	3.39
	**n**	6	6	6	6	6
**Group C - non-challenged**	**mean**	100.00	99.33	99.17	100.33	98.67
	**SD**	0.00	3.08	3.37	3.14	4.08
	**n**	6	6	6	6	6

Table S5: Two-way ANOVA of the mean body weight change between D0 and D4

ANOVA table	SS (Type III)	DF	MS	F (DFn, DFd)	P value
**Interaction**	311.8	20	15.59	F (20, 146) = 2.368	P=0.0018
**Row Factor**	987.8	4	246.9	F (4, 146) = 37.52	P<0.0001
**Column Factor**	487.7	5	97.55	F (5, 146) = 14.82	P<0.0001
**Residual**	960.9	146	6.582		

Bonferroni's multiple comparisons test	95.00% CI of diff.	Below threshold?	Summary	Adjusted P Value
**Group A vs. Group B**	-2.587 to 6.254	No	ns	>0.9999
**Group A vs. Group C**	-9.754 to -0.9131	Yes	**	**0.0065**
**Group B vs. Group C**	-11.59 to -2.746	Yes	****	**<0.0001**

Table S6: Viral RNA expression level (mean and SD) in lung from golden Syrian hamsters. Animals were randomized on D-3, inoculated with the viral strain on D0 and treated from D0 to D3. D4 was the last day of the study**.** Samples for qPCR were taken on D4. Results are expressed as $2^{-\Delta CT}$.

Group	n	Mean	SD	Minimum	Maximum
**Group A - RBD-IgY IN**	6	0.92	0.26	0.53	1.14
**Group B - non-treated**	6	2.86	1.33	1.15	4.27
**Group C - non-challenged**	6	0.02	0.01	0	0.04

Table S7: ANOVA of viral RNA expression level in lung from golden Syrian hamsters.

ANOVA table	SS	DF	MS	F (DFn, DFd)	P value
**Treatment (between columns)**	27.46	5	5.491	F (5, 29) = 6.606	**P=0.0003**
**Residual (within columns)**	24.10	29	0.8312		
**Total**	51.56	34			

Bonferroni's multiple comparisons test:

	Mean Diff.	95.00% CI of diff.	Summary	Adjusted P Value
**Groups A vs. B**	-1.945	-3.628 to -0.2616	*	**0.0136**
**Groups A vs. C**	0.9017	-0.7818 to 2.585	ns	>0.9999

Table S8: Viral load determined by $TCID_{50}$ (mL/g of lung, mean + SD) in lung from golden Syrian hamsters. Animals were randomized on D0, inoculated with the viral strain on D0 and treated from D0 to D3. D4 was the last day of the study**.**

Group	n	Mean	SD	Minimum	Maximum
**Group A - RBD-IgY IN**	6	2.17E+07	2.00E+07	1.97E+06	5.31E+07
**Group B - non-treated**	6	2.47E+07	1.73E+07	5.90E+06	5.31E+07
**Group C - non-challenged**	6	5.20E+02	0.00E+00	5.20E+02	5.20E+02

Table S9: ANOVA of viral load by TCID50 in lung from golden Syrian hamsters.

ANOVA table	SS	DF	MS	F (DFn, DFd)	P value
**Treatment (between columns)**	4.325e+015	5	8.651e+014	F (5, 29) = 2.207	P=0.0808
**Residual (within columns)**	1.137e+016	29	3.920e+014		
**Total**	1.569e+016	34			

Table S10: Summary of the lesion score in the lungs of golden Syrian hamsters. Animals were randomized on D-3, inoculated with the viral strain on D0 and treated from D0 to D3. D4 was the last day of the study.

Group	n	Mean	SD	Minimum	Maximum
**Group A - RBD-IgY IN**	6	3.083	0.5164	2.5	3.75
**Group B - non-treated**	6	2.292	0.2923	2	2.75
**Group C - non-challenged**	6	0.4583	0.4005	0	1

Table S11: ANOVA of the lesion scores in lung from golden Syrian hamsters.

ANOVA summary	
**F**	11.72
**P value**	**<0.0001**
**P value summary**	****
**Significant diff. among means (P < 0.05)?**	Yes
**R squared**	0.6690

Bonferroni's multiple comparisons test:

Bonferroni's multiple comparisons test	Mean Diff.	95.00% CI of diff.	Below threshold?	Summary	Adjusted P Value
**Groups A vs. B**	0.7917	-0.4009 to 1.984	No	ns	0.6360
**Groups A vs. C**	2.625	1.432 to 3.818	Yes	****	**<0.0001**
**Groups B vs. C**	1.833	0.6408 to 3.026	Yes	***	**0.0005**

Table S12: Quantitation of the cytokine expression levels in the lungs of golden Syrian hamsters expressed as $2^{-\Delta ct}$. Animals were randomized on D-3, infected with SARS-CoV-2 on D0 and treated from D0 to D3. D4 was the last day the study.

IFNγ:

Group	n	Mean	SD	Minimum	Maximum
Group A - RBD-IgY IN	6	1.29E-02	3.90E-03	8.11E-03	1.97E-02
Group B - non-treated	6	1.69E-02	3.83E-03	1.09E-02	2.16E-02
Group C - non-challenged	6	1.83E-03	6.45E-04	1.29E-03	2.80E-03

IL-2:

Group	n	Mean	SD	Minimum	Maximum
Group A - RBD-IgY IN	6	5.18E-04	2.48E-04	2.43E-04	8.79E-04
Group B - non-treated	6	2.25E-04	9.54E-05	1.24E-04	3.94E-04
Group C - non-challenged	6	3.07E-04	2.04E-04	1.20E-04	6.53E-04

IL-4:

Group	n	Mean	SD	Minimum	Maximum
Group A - RBD-IgY IN	6	2.19E-03	8.89E-04	9.38E-04	3.25E-03
Group B - non-treated	6	1.65E-03	1.07E-03	5.74E-04	2.92E-03
Group C - non-challenged	6	1.56E-03	7.91E-04	9.38E-04	2.99E-03

IL-6:

Group	n	Mean	SD	Minimum	Maximum
Group A - RBD-IgY IN	6	3.41E-03	1.69E-03	1.70E-03	6.28E-03
Group B - non-treated	6	3.85E-03	1.26E-03	1.89E-03	5.01E-03
Group C - non-challenged	6	2.03E-04	7.94E-05	1.06E-04	2.97E-04

IL-10:

Group	n	[illegible]	[illegible]	[illegible]	[illegible]
Group A - RBD-IgY IN	6	6.[illegible]	[illegible]	[illegible]	[illegible]
Group B - non-treated	6	1.[illegible]	[illegible]	[illegible]	[illegible]
Group C - non-challenged	6	1.[illegible]	[illegible]	[illegible]	[illegible]

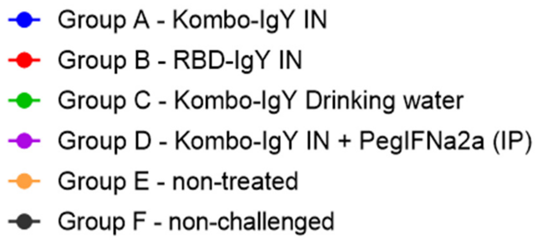

IL-12p40:

Group	n	Mean	SD	Minimum	Maximum
Group A - RBD-IgY IN	6	4.56E-04	1.89E-04	2.63E-04	7.53E-04
Group B - non-treated	6	2.66E-04	9.00E-05	1.57E-04	4.16E-04
Group C - non-challenged	6	2.05E-04	1.32E-04	1.04E-04	4.45E-04

IL-21:

Group	n	Mean	SD	Minimum	Maximum
**Group A - RBD-IgY IN**	6	1.53E-03	5.72E-04	9.35E-04	2.46E-03
**Group B - non-treated**	6	1.05E-03	3.64E-04	4.78E-04	1.45E-03
**Group C - non-challenged**	6	5.80E-04	4.89E-04	2.50E-04	1.55E-03

Table S13: ANOVA on the cytokine expression levels in the lungs of golden Syrian hamsters.

IFNγ:

ANOVA table	SS	DF	MS	F (DFn, DFd)	P value
**Treatment (between columns)**	0.001705	5	0.0003409	F (5, 29) = 11.05	**P<0.0001**
**Residual (within columns)**	0.0008947	29	3.085e-005		
**Total**	0.002599	34			

Bonferroni's multiple comparisons test	95.00% CI of diff.	Below threshold?	Summary	Adjusted P Value
**Groups A vs. B**	-0.01429 to 0.006224	No	ns	>0.9999
**Groups A vs. C**	0.0007680 to 0.02128	Yes	*	**0.0269**
**Groups B vs. C**	0.004800 to 0.02531	Yes	***	**0.0009**

IL-2:

ANOVA table	SS	DF	MS	F (DFn, DFd)	P value
**Treatment (between columns)**	3.761e-007	5	7.521e-008	F (5, 29) = 0.6374	P=0.6729
**Residual (within columns)**	3.422e-006	29	1.180e-007		
**Total**	3.798e-006	34			

IL-4:

ANOVA table	SS	DF	MS	F (DFn, DFd)	P value
**Treatment (between columns)**	6.349e-005	5	1.270e-005	F (5, 29) = 0.8160	P=0.5481
**Residual (within columns)**	0.0004513	29	1.556e-005		
**Total**	0.0005148	34			

IL-6:

ANOVA table	SS	DF	MS	F (DFn, DFd)	P value
**Treatment (between columns)**	7.412e-005	5	1.482e-005	F (5, 29) = 5.387	**P=0.0013**
**Residual (within columns)**	7.980e-005	29	2.752e-006		
**Total**	0.0001539	34			

Bonferroni's multiple comparisons test	95.00% CI of diff.	Below threshold?	Summary	Adjusted P Value
**Groups A vs. B**	-0.003499 to 0.002627	No	ns	>0.9999
**Groups A vs. C**	0.0001450 to 0.006271	Yes	*	**0.0339**
**Groups B vs. C**	0.0005810 to 0.006707	Yes	*	**0.0102**

IL-10:

ANOVA table	SS	DF	MS	F (DFn, DFd)	P value
**Treatment (between columns)**	0.0006354	5	0.0001271	F (5, 29) = 16.92	P<0.0001
**Residual (within columns)**	0.0002178	29	7.509e-006		
**Total**	0.0008532	34			

Bonferroni's multiple comparisons test	95.00% CI of diff.	Below threshold?	Summary	Adjusted P Value
**Groups A vs. B**	-0.009374 to 0.0007455	No	ns	0.1610
**Groups A vs. C**	0.001071 to 0.01119	Yes	**	**0.0084**
**Groups B vs. C**	0.005385 to 0.01551	Yes	****	**<0.0001**

IL-12p40:

ANOVA table	SS	DF	MS	F (DFn, DFd)	P value
**Treatment (between columns)**	3.980e-007	5	7.959e-008	F (5, 29) = 1.230	P=0.3205
**Residual (within columns)**	1.877e-006	29	6.471e-008		
**Total**	2.275e-006	34			

IL-21:

ANOVA table	SS	DF	MS	F (DFn, DFd)	P value
**Treatment (between columns)**	4.783e-006	5	9.565e-007	F (5, 29) = 2.080	P=0.0967
**Residual (within columns)**	1.334e-005	29	4.599e-007		
**Total**	1.812e-005	34			

Appendix A: Raw data

13 Raw Data List

Raw Data 1: Individual body weight (g) of golden Syrian hamsters infected with SARS-CoV-2 ____________36

Raw Data 2: Individual clinical scores and necropsy observations ____________________________________37

Raw Data 3: Individual lung qPCR results____38

Raw Data 4: Individual TCID50 results on lung tissue. ____39

Raw Data 5: Individual histopathological scoring of lung tissue.______________________________40

13.1 List of full group names

Group
**Group A - RBD-IgY IN**
**Group B - non-treated**
**Group C - non-challenged**

Raw Data 1: Individual body weight (g) of golden Syrian hamsters infected with SARS-CoV-2

			D-3	0	1	2	3	4
	Cage	Hamster #	16/07/2021	19/07/2021	20/07/2021	21/07/2021	22/07/2021	23/07/2021
**Group A**	**A1**	1	86	89	89	84	84	83
		2	98	105	103	100	97	96
		3	88	95	96	93	92	92
	**A2**	4	94	96	95	94	92	93
		5	90	93	93	91	85	84
		6	87	92	89	87	83	85
**Group B**	**B1**	7	85	93	88	83	81	79
		8	96	100	100	98	96	93
		9	97	102	101	99	97	96
	**B2**	10	90	94	91	90	92	86
		11	80	86	85	83	82	81
		12	96	105	102	97	96	97
**Group C**	**C1**	13	99	103	105	107	107	109
		14	88	92	91	90	89	89
		15	97	100	98	100	98	97
	**C2**	16	90	94	88	88	98	88
		17	91	94	95	92	92	94
		18	86	89	91	90	90	87

Raw Data 2: Individual clinical scores and necropsy observations

			D-3	0	1	2	3	4 / clinical signs	4 / necropsy
	Cage	Hamster #	16/07/2021	19/07/2021	20/07/2021	21/07/2021	22/07/2021	23/07/2021	23/07/2021
Group A	A1	1	Nothing to report	Nothing to report	Nothing to report	Nothing to report	Nothing to report	Nothing to report	Nothing to report
		2	Nothing to report	Nothing to report	Nothing to report	Nothing to report	Nothing to report	Nothing to report	Spots on the superior lobe & left lung
		3	Nothing to report	Nothing to report	Nothing to report	Nothing to report	Nothing to report	Nothing to report	Small spots on the lung
	A2	4	Nothing to report	Nothing to report	Nothing to report	Nothing to report	Nothing to report	Nothing to report	Nothing to report
		5	Nothing to report	Nothing to report	Nothing to report	Nothing to report	Nothing to report	Nothing to report	Nothing to report
		6	Nothing to report	Nothing to report	Nothing to report	Nothing to report	Nothing to report	Nothing to report	Spots on the PC,inferior & left lobe
Group B	B1	7	Nothing to report	Nothing to report	Nothing to report	Nothing to report	Nothing to report	Nothing to report	Nothing to report
		8	Nothing to report	Nothing to report	Nothing to report	Nothing to report	Nothing to report	Nothing to report	Nothing to report
		9	Nothing to report	Nothing to report	Nothing to report	Nothing to report	Nothing to report	Nothing to report	Nothing to report
	B2	10	Nothing to report	Nothing to report	Nothing to report	Nothing to report	Nothing to report	Nothing to report	Nothing to report
		11	Nothing to report	Nothing to report	Nothing to report	Nothing to report	Nothing to report	Nothing to report	Nothing to report
		12	Nothing to report	Nothing to report	Nothing to report	Nothing to report	Nothing to report	Nothing to report	Nothing to report
Group C	C1	13	Nothing to report	Nothing to report	Nothing to report	Nothing to report	Nothing to report	Nothing to report	Nothing to report
		14	Nothing to report	Nothing to report	Nothing to report	Nothing to report	Nothing to report	Nothing to report	Nothing to report
		15	Nothing to report	Nothing to report	Nothing to report	Nothing to report	Nothing to report	Nothing to report	Nothing to report
	C2	16	Nothing to report	Nothing to report	Nothing to report	Nothing to report	Nothing to report	Nothing to report	Nothing to report
		17	Nothing to report	Nothing to report	Nothing to report	Nothing to report	Nothing to report	Nothing to report	Nothing to report
		18	Nothing to report	Nothing to report	Nothing to report	Nothing to report	Nothing to report	Nothing to report	Nothing to report

Raw Data 3: Individual lung qPCR results

		CT									ΔCT								$2^{-\Delta CT}$							
	ID	ORF1ab	IFNg	IL-2	IL-4	IL-6	IL-10	IL-12p40	IL-21	g-Actin	ORF1ab	IFNg	IL-2	IL-4	IL-6	IL-10	IL-12p40	IL-21	ORF1ab	IFNg	IL-2	IL-4	IL-6	IL-10	IL-12p40	IL-21
Group A	1	23.467	24.942	30.431	28.228	27.148	26.253	30.617	28.786	22.562	0.905	6.218	11.707	9.504	8.424	7.529	11.893	10.062	0.534	0.013	0.000	0.001	0.003	0.005	0.000	0.001
	2	22.520	24.391	29.873	26.992	26.509	25.067	29.449	27.901	22.519	0.001	5.666	11.148	8.267	7.784	6.343	10.724	9.176	0.999	0.020	0.000	0.003	0.005	0.012	0.001	0.002
	3	22.671	25.383	29.240	27.535	27.653	26.825	29.463	27.755	22.859	-0.188	6.296	10.153	8.447	8.565	7.738	10.376	8.668	1.139	0.013	0.001	0.003	0.003	0.005	0.001	0.002
	4	22.561	25.297	29.109	27.557	27.407	26.622	29.994	27.844	22.625	-0.064	6.598	10.410	8.858	8.708	7.923	11.295	9.145	1.045	0.010	0.001	0.002	0.002	0.004	0.000	0.002
	5	21.547	24.208	29.930	27.984	25.239	24.808	29.726	27.733	21.719	-0.172	6.283	12.005	10.059	7.314	6.882	11.800	9.808	1.127	0.013	0.000	0.001	0.006	0.008	0.000	0.001
	6	23.456	25.951	29.944	27.605	28.205	27.510	30.119	28.777	22.865	0.591	6.947	10.939	8.601	9.200	8.505	11.115	9.772	0.664	0.008	0.001	0.003	0.002	0.003	0.000	0.001
Group B	7	21.533	23.631	30.743	28.325	26.313	24.092	29.954	27.689	21.736	-0.203	5.643	12.755	10.337	8.326	6.104	11.966	9.701	1.151	0.020	0.000	0.001	0.003	0.015	0.000	0.001
	8	19.986	23.837	30.343	29.070	25.952	24.381	30.489	27.956	22.082	-2.096	5.533	12.038	10.766	7.648	6.076	12.184	9.651	4.275	0.022	0.000	0.001	0.005	0.015	0.000	0.001
	9	21.116	24.468	29.884	27.398	26.306	25.166	29.806	28.004	22.418	-1.303	5.895	11.311	8.825	7.732	6.592	11.233	9.431	2.467	0.017	0.000	0.002	0.005	0.010	0.000	0.001
	10	21.730	25.069	31.284	27.641	27.430	25.967	30.847	28.929	23.504	-1.774	5.847	12.062	8.419	8.208	6.745	11.625	9.707	3.419	0.017	0.000	0.003	0.003	0.009	0.000	0.001
	11	23.050	26.499	32.954	28.525	29.026	27.447	31.994	31.006	23.739	-0.688	6.524	12.979	8.549	9.050	7.471	12.019	11.030	1.612	0.011	0.000	0.003	0.002	0.006	0.000	0.000
	12	20.061	24.349	30.445	28.650	25.899	25.058	30.889	28.653	22.151	-2.090	6.092	12.188	10.394	7.642	6.801	12.633	10.396	4.257	0.015	0.000	0.001	0.005	0.009	0.000	0.001
Group C	13	26.975	28.145	31.268	28.511	30.313	31.436	31.818	30.473	22.373	4.602	9.552	12.674	9.918	11.720	12.843	13.224	11.879	0.041	0.001	0.000	0.001	0.000	0.000	0.000	0.000
	14	31.575	28.515	31.945	28.558	32.118	32.906	31.983	30.880	22.572	9.003	9.601	13.030	9.643	13.203	13.991	13.068	11.965	0.002	0.001	0.000	0.001	0.000	0.000	0.000	0.000
	15	28.601	27.653	31.432	27.972	31.682	31.085	31.837	30.263	22.813	5.788	8.665	12.445	8.984	12.694	12.098	12.849	11.275	0.018	0.002	0.000	0.002	0.000	0.000	0.000	0.000
	16	28.119	28.340	30.075	28.930	30.587	30.749	31.536	29.721	22.506	5.614	9.469	11.203	10.058	11.715	11.877	12.664	10.849	0.020	0.001	0.000	0.001	0.000	0.000	0.000	0.001
	17	29.700	27.825	29.927	27.731	31.583	31.813	30.480	28.678	23.364	6.336	8.480	10.582	8.386	12.238	12.468	11.135	9.333	0.012	0.003	0.001	0.003	0.000	0.000	0.000	0.002
	18	29.200	28.199	30.637	28.728	31.602	32.074	30.826	30.036	22.816	6.384	9.213	11.651	9.742	12.616	13.089	11.840	11.051	0.012	0.002	0.000	0.001	0.000	0.000	0.000	0.000

Raw Data 4: Individual TCID50 results on lung tissue.

Group	#ID	Log10 TCID50/mL	Log10 TCID50/48h/mL/g lung	TCID50 mL/g lung
A	H01	6.49	7.49	30682760
	H02	5.29	6.29	1968300
	H03	6.49	7.49	30682760
	H04	6.73	7.73	53144100
	H05	5.53	6.53	3409196
	H06	6.01	7.01	10227587
B	H07	6.73	7.73	53144100
	H08	6.49	7.49	30682760
	H09	5.77	6.77	5904900
	H10	6.49	7.49	30682760
	H11	6.01	7.01	10227587
	H12	6.25	7.25	17714700
C	H13	1.72	2.72	520
	H14	1.72	2.72	520
	H15	1.72	2.72	520
	H16	1.72	2.72	520
	H17	1.72	2.72	520
	H18	1.72	2.72	520

Raw Data 5: Individual histopathological scoring of lung tissue.

		Slice 1			SUM	Slice 2			SUM	Slice 3			SUM	Slice 4			SUM
Group	Hamster #	Inflammation	Edema	Hemorrhage		Inflammation	Edema	Hemorrhage		Inflammation	Edema	Hemorrhage		Inflammation	Edema	Hemorrhage	
Group A	1	2	1	1	**4**	2	1	1	**4**	1	1	1	**3**	1	1	0	**2**
	2	2	1	0	**3**	2	1	0	**3**	1	1	0	**2**	1	1	0	**2**
	3	1	1	1	**3**	2	1	1	**4**	2	1	1	**4**	1	1	1	**3**
	4	1	0	0	**1**	1	1	1	**3**	1	1	1	**3**	1	1	1	**3**
	5	2	1	1	**4**	2	1	1	**4**	1	1	1	**3**	2	1	1	**4**
	6	1	1	0	**2**	2	1	1	**4**	2	1	1	**4**	1	1	0	**2**
Group B	7	2	1	0	**3**	2	1	1	**4**	1	1	0	**2**	1	1	0	**2**
	8	2	1	0	**3**	2	1	0	**3**	1	1	0	**2**	1	1	0	**2**
	9	1	1	0	**2**	2	1	0	**3**	1	1	0	**2**	1	1	0	**2**
	10	1	1	0	**2**	1	1	0	**2**	2	1	0	**3**	1	1	0	**2**
	11	1	1	0	**2**	1	1	0	**2**	1	1	0	**2**	1	1	0	**2**
	12	1	1	0	**2**	1	1	0	**2**	1	1	0	**2**	1	1	0	**2**
Group C	13	0	1	1	**2**	0	0	0	**0**	0	0	0	**0**	0	0	0	**0**
	14	0	0	0	**0**	0	0	0	**0**	0	0	0	**0**	0	0	0	**0**
	15	0	1	0	**1**	0	0	0	**0**	0	0	0	**0**	0	1	0	**1**
	16	0	0	0	**0**	0	0	0	**0**	0	0	0	**0**	0	0	0	**0**
	17	0	1	1	**2**	0	1	0	**1**	0	0	0	**0**	0	0	0	**0**
	18	0	1	0	**1**	0	1	0	**1**	0	1	0	**1**	0	1	0	**1**

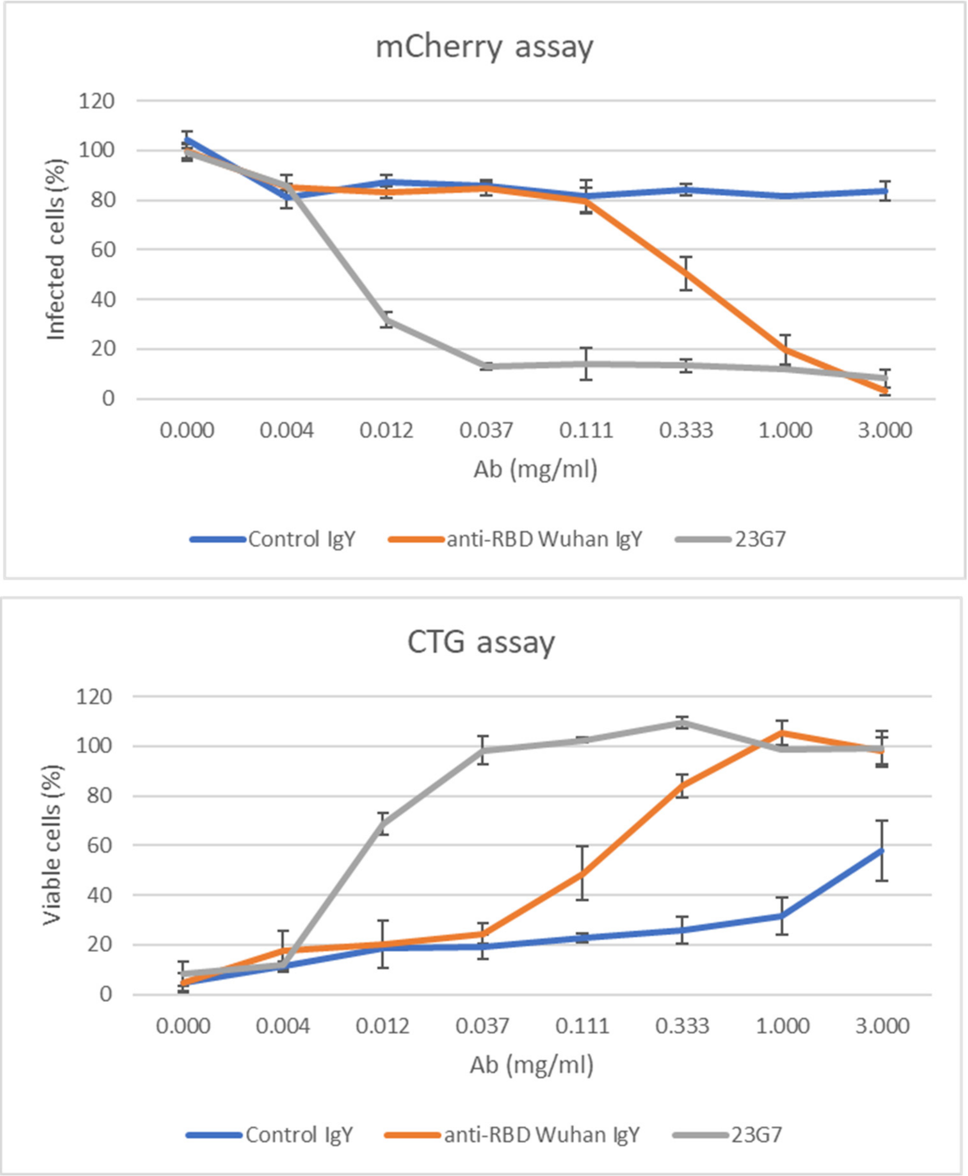

Figure S1. Comparison of neutralization capacity of anti-RBD Wuhan antibodies produced in chicken eggs and human monoclonal 23G7 antibody. (**a**) Neutralization capacity of anti-RBD_Wuhan IgY, 23G7 and control non-immune IgYs was measured by monitoring mCherry fluorescence of Vero-E6 cells after 48 h of infection with mCherry-encoded SARS-CoV-2/Wuhan. Mean; n = 3. (**b**) Neutralization capacity of anti-RBD_Wuhan, 23G7 and control non-immune IgYs was measured by monitoring viability of Vero-E6 cells after 48 h of infection with mCherry-encoded SARS-CoV-2/Wuhan. Mean; n = 3.
